# Organisational Justice, Burnout, and Engagement in University Students: A Comparison between Stressful Aspects of Labour and University Organisation

**DOI:** 10.3390/ijerph15102116

**Published:** 2018-09-26

**Authors:** Yolanda Navarro-Abal, Juan Gómez-Salgado, María José López-López, José Antonio Climent-Rodríguez

**Affiliations:** 1 Department of Social, Evolutionary and Educational Psychology, University of Huelva, 21071 Huelva, Spain; yolanda.navarro@dpsi.uhu.es (Y.N.-A.); jose.climent@dpsi.uhu.es (J.A.C.-R.); 2 Department of Nursing, University of Huelva, 21071 Huelva, Spain; 3 Espíritu Santo University, Guayaquil 092301, Ecuador; 4 Department of Clinical and Experimental Psychology, University of Huelva, 21071 Huelva, Spain; mjlopez@uhu.es

**Keywords:** academic well-being, burnout, academic engagement, students, organisational justice, stress

## Abstract

Burnout, engagement, and organisational justice concepts are usually studied in the context of labour organisations, but not in universities. For this, the objective of this research is to identify the students’ empirically evidenced relationships in the employment context, such as levels of organisational justice, stress indicators, burnout and work commitment. On the other hand, engagement is analysed as a mediating variable that explains the relationship between organisational justice and burnout. A sample of 543 students from three Spanish universities, selected by purposive sampling, is used ensuring voluntary and anonymous participation. The instruments used to measure the four variables to analyse are a protocol for data collection, MBI-SS instrument for Academic Burnout, Utrecht Work Engagement Student Scale (UWES) for Engagement and the Scale of Organisational Justice for Organisational Justice. As a result, college students show behaviours that promote academic achievement, and they feel more engaged when they are treated fairly. As for the burnout syndrome dimensions, average levels of emotional exhaustion and academic efficacy, and high levels of cynicism are revealed. In addition, the proposed structural equation model supports the main hypothesis; engagement is a mediating variable in the organisational justice and burnout relationship. To conclude, academic stress and its explanatory framework cannot be conceived only from an organisational perspective, where the context of each student must be considered. The adoption of organisational preventive measures can be relevant in ensuring a healthy and conducive academic performance in our students.

## 1. Introduction

### 1.1. Burnout Syndrome and Its Incidence at the University Level

The burnout syndrome is internationally acknowledged as one of the few syndromes, which, despite its relationship with mental disorders, has minimal stigma attached to it [1]. The explanation of its aetiology is attributed to many variables related to the background and to socio-professional causes, being the most common “environment” and “work context”. At the same time, few variables are related to people, thus freeing them from stigmatization.

The syndrome consists of three stages called “emotional fatigue” (EF), “depersonalization” (DP), and “lack of personal accomplishment”. When adapting them to the academic context, they are specified as “emotional exhaustion”, “cynicism”, and “academic efficacy” [2]:

- Emotional exhaustion (EE): characterized by feeling exhausted by the activity demands.
- Depersonalization (DP)/cynicism: the attitude of coldness and distancing in interpersonal relationships.
- Personal Accomplishment (PA)/academic efficacy: defined as feelings of lack of self-efficacy and personal fulfilment at work.

According to Schaufeli et al. [3], there are three types of theoretical models that attempt to explain this syndrome, each emphasizing the influence of individual [4], social [5], and organisational variables [6]. Navarro-Abal et al. [7] point out the importance of integrating the three perspectives as the only model that encompasses enough variables to explain this syndrome. Following the Demand-Control model base by Karasek, explaining occupational stress and burnout, a wider view is given by adding the social support dimension. The basic hypothesis claims that highly demanding job positions and little control, together with little social support in the workplace, imply a higher risk of suffering burnout [8].

One line of study focuses on the personality characteristics [9]. Colino and Pérez de León [10] collect studies that explain the importance of the pattern of Type A behaviour as a way of unhealthy and maladaptive coping, while Moreno-Jiménez, Garrosa, Corso et al. [11] highlight the lack of appropriate standards, such as the so-called mental strength or toughness.

Although, initially, this syndrome affects professionals who provide their service in direct personal care, [12] more contemporary works approach the issue from a much broader perspective, relating it to other professional groups [13].

### 1.2. Burnout in University Students

Generically, academic burnout could be defined as that which develops in relation to educational institutions and that may affect either teachers or students at any educational level [14].

As it occurs with professionals, it is observed that, students who began their studies with enthusiasm, subsequently came to express a sense of disappointment, lack of energy, fatigue, feeling of emptiness or failure, low self-esteem, lack of concentration, and desire to leave their studies [15].

Thus, in recent years, there has been a growing interest in academic stress or burnout. Several authors have studied how students find themselves exhausted by the demands of their studies, developing a cynical attitude of detachment and feelings of inadequacy as a student [16].

Burnout among university students has the same characteristics as burnout in the professional field [17]. However, emotional exhaustion is highlighted as the most prevalent symptom in relation to the other two dimensions.

### 1.3. Engagement in University Students

The latest trends in psychology studies reflect an increase of scientific studies that are related with the Positive Psychology line of work, and the importance of people’s positive aspects and strengths is emphasised, rather than the pathological and dysfunctional aspects [18].

Shaufeli [19] identifies three psychological conditions that engaged people show: satisfaction, security and availability. Currently, engagement is analysed from two different occupational perspectives: the role theory and occupational health. Both agree that this process integrates three components:

- Vigour (behavioural-energy component, as opposed to emotional exhaustion).
- Absorption (cognitive component, as opposed to personal fulfilment/academic efficacy).
- Dedication (emotional component, as opposed to cynicism).

“Engaged students” is a term used to represent the willingness of students to participate in their daily activities, such as attending classes, working independently, participating in practical activities and following teachers’ instructions in academic activities [20]. These students usually take part in the teaching-learning process with a positive attitude [21], as characterized by enthusiasm, optimism, curiosity, and interest in tasks selected according to their competence level, taking advantage of the offered opportunities, making an intense effort and concentrating on learning tasks [21].

Another study by Kenney, Dumont and Kenney [22] identified five indicators for university students’ participation. These included the level of academic challenge, an active and collaborative attitude in the teaching-learning process, a high level of commitment and involvement of university students in the organisation, participating and enriching the working environment. Commitment is increasingly perceived as an indicator of success in academic performance.

### 1.4. Organisational Justice

The term “organisational justice” was coined in 1987 by Greenberg, defining it as the employees’ perception of what they consider fair in the organisation. It has been the subject of multiple theories that have attempted to describe its characteristics. For example, Rodriguez et al. [23] report the Adams’ Equity theory (1965), the Homans’ version of the Social Exchange theory (1961), Blau’s Social Exchange theory about expectations (1964), the Multiple Distribution rules by Leventahal (1976), Thibaut and Walker’s Procedural Fairness in Dispute Resolution theory (1975), and Bies and Moag’s Interpersonal Treatment theory (1986). Colquitt [24] integrates the various theories on justice by developing a model of organisational justice consisting on four dimensions: distributive justice, procedural justice, interpersonal justice, and informational justice.

Other studies show that organisational justice is negatively related with the intentions of abandonment [25], burnout [26], and psychological stress [27]. Similarly, there is evidence that proves that organisational justice is positively related to job satisfaction, organisational trust and support [28], and work engagement [29].

Skholer and Tziner [30] analyse different research on the relationship between burnout and organisational justice perception, concluding that, although research on both variables is scarce and the theoretical rationalisation to link both constructs is vague, there is a general tendency towards the perception of organisational justice in relation to the burnout level.

The main aim of this paper is to analyse those empirically evidenced relations in the employment context while focusing on the academic setting, studying the different dimensions of organisational justice in terms of the levels of burnout and engagement.

Taking into account the findings mentioned above, the following hypothesis arises in this study: the relationship between organisational justice (procedural, interpersonal, informational, and distributive) and burnout (emotional exhaustion, cynicism, and academic efficacy) is mediated by engagement (dedication, vigour, and absorption).

## 2. Materials and Methods

The online version of the assessment instruments used in this study was developed through the Google Docs tool, together with information on the study objectives and the pertinent informed consent. Then, the electronic link to this version was sent by e-mail to four teachers from the University of Huelva, two from the University of Seville and two from the Complutense University of Madrid, asking them to disseminate it among their students through the Moodle online learning platform. Students who voluntarily agreed to collaborate in the research had access to complete the form between 20 April and 30 May 2017, coinciding with the final period of the second semester and before the start of the final exam period. This decision was based on previous experience during which it was observed that the longer the time allowed for the completion of evaluation tests, the lower the response rate, and the higher the probability of “central tendency error” bias.

### 2.1. Setting and Participants

The sample consisted of 543 students from the University of Huelva, the Complutense University of Madrid and the University of Seville.

39.5% of the subjects were men (*n* = 214) and 60.5% were women (*n* = 328). Although this is an imbalanced sampling regarding sex, it is representative of the actual Spanish university population, characterised by a greater presence of women. According to the age variable, it ranged between 19 and 55 years of age (M = 23.6, SD = 6.20).

Regarding the distribution by course, the data show that 28.7% were first-year students, 37.1% second, 13.7% third, and 20.5% fourth-year. They were enrolled in the following degrees: 213 in Psychology (39.2%), 116 in Labour Relations and Human Resources (21.3%), 47 in Computer Engineering (8.6%), 50 in Law (9.2%), and 117 in Primary Education (21.7%).

The sample selection was conducted by purposive sampling. With regard to sample size, as Hox, Moerbeek, and Van De Schoot point out [31], a ratio of at least ten times the number of cases over the number of variables was considered, i.e., an observation/item ratio of 10:1, as a necessary requirement to carry out an appropriate factor analysis. Thus, as the total number of items used in the assessment tools is 52, it is estimated that the sample size must be of a minimum of 520 participants.

### 2.2. Instruments and Variables

Four types of variables have been considered. The instruments used were the following:

(a) Data collection protocol. Different demographic variables were collected (age, sex, degree, course), along with other variables of interest (reason for choosing the degree, fulfilling expectations regarding the degree, teacher training review, and expectations of improving employability).
(b) Academic burnout. The MBI-SS instrument was used [16], consisting of 15 items comprising three dimensions: emotional exhaustion (five items), cynicism (four items), and academic efficacy (six items). The answers follow a Likert-type format with seven possible answers from never/never once (0) to always/every day (6). The highest degree of burnout occurs when high scores are indicated in the dimensions of emotional exhaustion and cynicism, and low scores on academic efficacy.
(c) Engagement. The instrument used was the “Utrecht Work Engagement Student Scale (UWES)”, consisting of 17 items divided into three subscales: vigour (six items), dedication (five items), and absorption (six items) [32]. The answers follow a Likert-type format with seven possible answers ranging from never/never once (0) to always/every day (6). The assessment of this questionnaire moves in the right direction, so that the higher the score, the higher the levels of vigour, dedication and absorption.
(d) Organisational justice. The Organisational Justice scale has been used [24], consisting of 20 items divided into four subscales: distributive justice (JD) (four items), procedural justice (JP) (seven items), interpersonal justice (JINT) (four items), and informational justice (JINF) (five items). Responses follow a Likert-type format with 5 possible answers ranging from 1 (none) to 5 (much/a lot). Higher scores indicate a higher level of perception of organisational justice.

## 3. Results

This section presents the results obtained in the different statistical analyses performed. Structural equations modelling (SEM) are proposed, using a multivariate statistical analysis that allows for proposing causal relationships among the variables under study.

Table 1 presents the means, standard deviations, Cronbach’s alphas, and intercorrelations of the relevant variables in this study.

General values in the burnout syndrome dimensions revealed, on the one hand, close to average levels in the emotional exhaustion (M = 2.68, SD = 1.02) and academic efficacy (M = 2.90, SD = 0.92) dimensions, and, secondly, high levels in the cynicism dimension (M = 4.60, SD = 0.74). These results indicate that university students showed some emotional fatigue and perceived moderate academic efficacy, but nevertheless presented feelings of coldness and detachment in interpersonal relationships, as characterised by passivity towards teachers’ demands and disruptive behaviour in the classroom, as well as absenteeism, in the academic sphere.

In relation to the general values of engagement, the observed levels are below the average in the three dimensions: vigour (M = 2.50, SD = 1.20), dedication (M = 2.30; = 0.80), and absorption (M = 2.40; DT = 0.43). In general, one could say that students showed little enthusiasm or commitment, failed to seek challenges, and found little satisfaction in the work done.

As for the general values of the organisational justice dimensions, low levels are found in distributive (M = 1.9, SD = 0.70) and procedural justice (M = 1.7, SD = 0.22), and levels substantially below the average in interpersonal (M = 2.4, SD = 0.64) and informational justice (M = 2.6, SD = 0.83). On the other hand, regarding the way that they feel treated and the feedback they perceive from their teachers and management teams in relation to their decisions, they do not consider these attitudes as very appropriate, although they could be acceptable. All intercorrelations are significant, ranging between 0.21 and 0.86.

In relation to the academic burnout syndrome and organisational justice dimensions, the dimensions of emotional exhaustion and cynicism show a negative correlation with all of the organisational justice dimensions (distributive justice (*r* = −0.86; *p* < 0.001); procedural justice (*r* = −0.51; *p* < 0.001); interpersonal justice (*r* = −0.46; *p* < 0.001); and, informational justice (*r* = −0.51; *p* < 0.001). This same trend is observed between cynicism and all the organisational justice dimensions (distributive justice (*r* = −0.21; *p* < 0.05); procedural justice (*r* = −0.58; *p* < 0.05), interpersonal justice (*r* = −0.79; *p* < 0.05) and informational justice (*r* = −0.59; *p* < 0.05), whereas the dimension of academic efficacy indicates positive correlations (distributive justice (*r* = 0.58; *p* < 0.001); justice procedural (*r* = 0.41; *p* < 0.001); interpersonal justice (*r* = 0.46; *p* < 0.001); and, informational justice (*r* = 0.39; *p* < 0.001)). In other words, the lower the levels of the organisational justice perceived, the greater the sense of exhaustion and the feelings of coldness. Moreover, it is observed that the lower the feeling of injustice, the greater the perception of academic efficacy.

As for engagement, we can see that all of the dimensions, vigour (distributive justice (*r* = 0.38; *p* < 0.001); procedural justice (*r* = 0.21; *p* < 0.001); interpersonal justice (*r* = 0.48; *p* < 0.001) and informational justice (*r* = 0.37; *p* < 0.001), dedication (distributive justice (*r* = 0.33; *p* < 0.001); procedural justice (*r* = 0.46; *p* < 0.001); interpersonal justice (*r* = 0.75; *p* < 0.001) and informational justice (*r* = 0.48; *p* < 0.001)), and absorption (distributive justice (*r* = 0.21; *p* < 0.05), procedural justice (*r* = 0.77; *p* < 0.05), interpersonal justice (*r* = 0.49; *p* < 0.05), and informational justice (*r* = 0.34; *p* < 0.05)) show a positive correlation with all of the organisational justice dimensions. That is, the greater the sense of justice, the greater the vigour, dedication, and absorption towards the performed task.

However, the correlation that was established between emotional exhaustion and distributive justice (*r* = −0.86; *p* < 0.001), cynicism and interpersonal justice (*r* = −0.79; *p* < 0.001), dedication and interpersonal justice (*r* = 0.75; *p* < 0.001), and, finally, absorption and procedural justice (*r* = 0.77; *p* < 0.05) should be noted for its high value. Now, the correlations between the underlying factors are shown in Table 2, ranging from 0.79 to 0.82.

The goodness of fit of the proposed model was assessed while using the goodness of fit index (GFI), getting a result of 0.89, which, according to the recommendations made by Ruiz, Pardo, and San Martín [33], is considered to be a good level of adjustment to the model. In addition, the adjusted goodness of fit index (AGFI) gave a result of 0.93, concluding that the model is satisfactory.

### Hypothesis Model

For the structural equation model approach, all of the dimensions of organisational justice, engagement and burnout were taken into account as underlying variables. As Colquitt [34] points out, organisational justice is considered as a second order variable, formed by four dimensions (distributive, interpersonal, informational and procedural). In relation to the engagement construct, Schaufeli, Bakker, and Salanova [35] describe the same but consisting of three dimensions (vigour, dedication, and absorption). Finally, Maslach, Schaufeli and Leiter [2] offer burnout as a three-factor construct (emotional exhaustion, cynicism and academic effectiveness).

The model that is proposed in this paper reveals that the relationship between organisational justice and burnout is mediated by engagement. Then, the simple mediation procedure arose in order to analyse the mediating variable effect in the relationship between independent or explanatory variables and other dependent or explained variables. This procedure is also called “analysis of direct and indirect effects”. The most frequent procedure to test the variables mediating in a research was developed by Baron and Kenny [36] and consists of four stages that involve a three regression equations estimation. Depending on the model, it is necessary to meet the following conditions in order to analyse the mediation: (1) the three variables, predictive variable (PV), measuring variable (MV), and criteria variable (CV), are significantly interrelated; (2) by including the modulating variable, the relationship between the predictive variable (PV) and the criteria (CV) decrease; and, (3) when considering both variables jointly, i.e., PV and MV, the relationship of the latter (MV) with CV remains significant.

**Figure 1:**
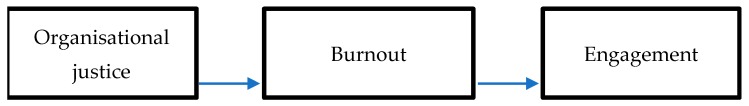
Mediating model (mediated or indirect causal relationship).

The scheme that is proposed to represent this study hypothesis of mediation is described in Figure 1, according to which the effect of the predictive variable (organisational justice) on the criteria (burnout) is established by the influence of the mediating variable effect (engagement).

The results obtained after the mediation process were (Table 3):

In the first stage, there was a significant negative relationship between organisational justice and burnout (β = −0.51; *p* < 0.001); in the second stage, organisational justice showed a significant and positive relationship with engagement (β = 0.48; *p* < 0.001); in the third stage, the results indicate a significant negative relationship between engagement and burnout (β = −0.32; *p* < 0.001), being the organisational justice variable constant. Finally, in the fourth stage, the partial mediation model was tested, and the coefficient of regression between organisational justice and burnout was significant and negative (β = −0.36; *p* < 0.001).

**Figure 2:**
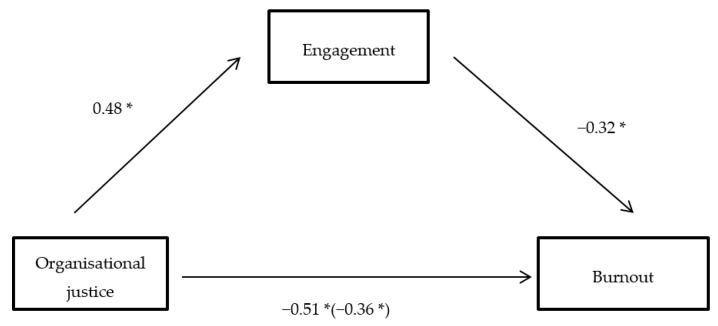
Engagement mediating effect between the organisational justice and burnout variables. (*) *p* < 0.001; (c) [beta] is obtained from the analysis, considering both organisational justice and engagement.

According to the obtained data, the relationship between organisational justice and burnout is partially mediated by the engagement dimensions. Therefore, we claim that the variable is mediated, since the mediating variable has a significant beta (β = 0.32; *p* < 0.001). In addition, this is a partial mediation, since, although the organisational justice beta has been reduced (from 0.51 to 0.36), it is still significant. These results indicate that part of the association between organisational justice and burnout is explained because organisational justice is associated with a higher level of engagement, leading to perceive less burnout. Also, as Calvete and Gale [37] indicate, these results show that there are other ways by which the PV can influence the CV. Finally, following some authors’ recommendations, such as those by Kenny, Kashy, and Bolger [38], in order to verify that the obtained mediation is statistically significant, the Sobel test was applied. In this case, the obtained mediation is statistically significant (Z = 2.94, *p* < 0.001). Therefore, the results support the proposed hypothesis. In Figure 2, there is a graphic representation of the proposed model.

## 4. Discussion

As evidenced in previous research within employment contexts [39], the present study found very similar results when applied to the academic context of Spanish universities. Thus, Spanish university students are more likely to feel comfortable with the degree that they are studying and the university institution if they value positively the way they are treated [34]. As mentioned at the beginning of this study, organisational justice is positively and negatively associated with different variables that influence people’s behaviour and attitudes, and burnout [26] and engagement [29] act as two indicators of both trends.

The results that are shown in this study prove the positive interdependence relationship between organisational justice and engagement level, as well as the negative interdependence relationship between organisational justice and burnout levels among the university students who participated in the study. Likewise, the proposed hypothesis of engagement as a mediating variable in the relationship between organisational justice and burnout has been proven, confirming the proposed hypothesis.

The established relationships between organisational justice and engagement included a highly significant presence in the four dimensions (procedural, distributive, interpersonal and informational justice). In the case of the last two dimensions (interpersonal and informational), an explanatory analysis could be established through the Social Exchange theory, as proposed by Blau in 1964.

On the other hand, it is understood that the Adams’ Equity theory provides the basis to explain how perceptions of the other two studied dimensions of organisational justice (distributive and procedural), based on instrumental and economic resources (such as rewards and/or bonuses), also influence students’ engagement and external motivators [40]. Trechera [41] adds that, in order to establish that criterion, the person considers two types of elements: inputs, in the form of contributions that the student brings to the task, and outputs, as the results, advantages, and benefits that the student obtains for the academic work done. This theory is important insofar as it considers the critical students’ perception of what they are doing, or intend to do, as fundamental [42].

In recent years, under the new stream of positive psychology, studies have focused on strengths and performance and on the flow theory [43]. Thus, tools have been developed to assess concepts, such as engagement, totally opposed to burnout. Similarly, one could interpret that the more fairly treated students that feel by their institution, peers and teachers, the less likely they are to experience emotional exhaustion, depersonalisation and cynical attitudes, as well as lack of academic efficacy [32]. Following this approach, the Conservation of Resources theory develops the idea that one aims at obtaining and conserving resources, both personal and social, experimenting psychological stress and burnout before unfair situations within one’s organisation, thus being these negative results a menace for the loss of resources [44].

In terms of specific dimensions, Cole et al. [45] showed that distributive and interpersonal justice are negatively related to emotional exhaustion, while procedural justice did not predict it. However, Yildirim et al. [46] reported that levels of distributive, procedural, and interpersonal justice predict emotional exhaustion and depersonalisation among teachers and management staff. In our study, it is clearly seen how interpersonal justice appears as a consistent predictor of burnout, especially associated with the students’ attitudes of cynicism and depersonalisation, as corroborated by later studies [47]. Procedures and forms of unfair treatment may indicate the students’ lack of control and social exclusion situation within their organisation. The findings of longitudinal studies, such as those by Kivimäki et al. [48], confirm that unfair treatment is one of the biggest sources of job stress and it increases the probability of physical health hazards.

Another relation found in our study predicts the inverse relationship between distributive justice and students’ emotional exhaustion. The above findings have shown that people’s attitudes and emotional reactions are strongly influenced by organisational justice [49]. It also confirms previous findings in this sense, using perceived job insecurity as a moderating variable [50].

Finally, note the important relationship found between the level of procedural justice and the absorption factor, suggesting that students will manifest higher levels of concentration and happiness during their academic activity if they perceive that their university institution is governed by clear, acceptable procedures.

### 4.1. Limitations

The main limitation of this study is that it is a cross-sectional and descriptive research, in which the data pertain to a specific point in time, and conclusions cannot be drawn about the aetiology or causation of the obtained results. However, the great advantage of this design is that it identifies a measure of engagement and burnout perceptions and their possible relation to how organisational justice is perceived in a segment of the university’s student body. This will allow for an analysis of the possible variables to be proposed in preventive initiatives to help improve the well-being and, consequently, the teaching-learning processes, favouring better training and academic performance.

### 4.2. Implications for School Health

The results indicate that, when university students feel that they are treated fairly, they show behaviours that promote academic achievement, and feel more engaged with their academic work. Similarly, according to the outcomes, levels of burnout can influence real and perceived student performance, in terms of the opportunity to finish their education properly and enter the labour market successfully. Although it is clear that academic stress and its explanatory and intervention framework cannot be conceived from an organisational perspective alone, as personal variables and the context of each student also exert an influence, the adoption of preventive measures in universities at the organisational level may be conducive to ensuring healthy and ongoing academic performance in our students.

## 5. Conclusions

While this study seeks to identify the relationships, empirically evidenced in the employment context, among the basic levels of organisational justice and related academic stress indicators including burnout and students’ commitment to their tasks, it is presented as a framework of interest that allows for a better understanding of the relationships between organisational behaviour, stress, and academic performance in the Spanish university scope. In this sense, we believe that it necessary to highlight that the sex distribution used in this study sample, mostly women, reflects the present situation of the Spanish university system, as characterized by a greater presence of women, both among enrolled and graduated students, as the last published statistics show [51,52].

Although it has already been amply demonstrated in employment contexts, this study shows that when university students feel they are treated fairly, they not only present behaviours that favour their academic performance, but also feel more engaged with their tasks, being more committed to their studies and more proactive and participatory in university life. Engaged students are associated with various facilitators of academic activity, such as greater happiness and greater satisfaction in relation to their tasks and are less likely to drop out. Similarly, burnout levels can influence real and perceived students’ performance.

In this regard, the adoption of preventive measures at the organisational level in universities may help to ensure healthy and encouraging academic performance in our students. It seems more reasonable to think in terms of holistic approaches that allow the detection and modification of the stressful aspects of current educational organisations and systems.

## Figures and Tables

**Table 1:** Means (M), Standard Deviations (SD) and Intercorrelations between variables (*N* = 543).

Variables	M	SD	1	2	3	4	5	6	7	8	9
1. Emotional exhaustion	2.68	1.02	−								
2. Cynicism	4.60	0.74	0.59 **	−							
3. Academic efficacy	2.90	0.92	−0.41 **	−0.23 *	-						
4. Vigour	2.50	1.20	−0.66 **	−0.42 **	0.59 **	-					
5. Dedication	2.30	0.80	−0.59 **	−0.54 **	0.64 **	0.54 **	-				
6. Absorption	2.40	0.43	−0.36 *	−0.75 **	0.53 **	0.44 **	0.49 **	-			
7. Distributive Justice	1.9	0.70	−0.86 **	−0.21 *	0.58 **	0.38 **	0.33 **	0.21 *	-		
8. Procedural Justice	1.7	0.22	−0.51 **	−0.58 *	0.41 **	0.21 **	0.46 **	0.77 *	0.58 **	-	
9. Interpersonal Justice	2.4	0.64	−0.46 **	−0.79 *	0.46 **	0.48 **	0.75 **	0.49 *	0.56 **	0.66 **	-
10. Informational Justice	2.6	0.83	−0.51 **	−0.59 *	0.39 **	0.37 **	0.48 **	0.34 *	0.44 **	0.53 **	0.55 **

Notes: ** *p* < 0.001; * *p* < 0.05.

**Table 2:** Correlations between underlying factors, composite reliability coefficient and average variance extracted.

	CRC	AVE	1	2	3
Organisational Justice	0.87	0.61	-		
Engagement	0.91	0.84	0.88	-	
Burnout	0.83	0.63	0.79	0.82	-

Notes: CRC = Composite Reliability Coefficient; AVE: Average Variance Extracted.

**Table 3:** Analysis of engagement as mediating variable in the relationship between organisational justice and burnout.

Predictor	B	Standard Error	β	t	F	R^2^	Criteria
Step I. PV on CV Organisational Justice (VI)	−1.52	0.14	−0.51	−5.89 *	48.65 *	0.24	*Burnout*
Step 2. PV on MV Organisational Justice (VI)	0.91	0.22	0.48	6.32 *	39.41 *	0.22	*Engagement*
Steps 3 and 4. PV and MV on CV Organisational Justice (VI)	−1.19	0.19	−0.36	−4.04 *	39.38 *	0.33	*Burnout*
*Engagement* (M)	−0.57	0.09	−0.32	−4.25 *			

Notes: * p < 0.001; *N* = 543.
